# z‐VAD‐FMK triggers macrophage necroptosis during mycobacterial infection via a non‐canonical off‐target mechanism

**DOI:** 10.1002/mlf2.70101

**Published:** 2026-10-01

**Authors:** Rong Hu, Pei Li, Jie Han, Jiamin Huang, Guoyi Huang, Xiuju Jiang, Daniel Pfau, Guobao Li, Yi‐Nan Gong, Carl F. Nathan, Li Zhang

**Affiliations:** ^1^ Department of Immunology and Microbiology, School of Life Sciences Southern University of Science and Technology Shenzhen China; ^2^ Shenzhen Third People's Hospital The Second Affiliated Hospital of Southern University of Science and Technology Shenzhen China; ^3^ Department of Microbiology and Immunology Weill Cornell Medicine New York New York USA; ^4^ Department of Immunology University of Pittsburgh School of Medicine Pittsburgh Pennsylvania USA; ^5^ Tumor Microenvironment Center, UPMC Hillman Cancer Center University of Pittsburgh Pittsburgh Pennsylvania USA; ^6^ Cancer Immunology and Immunotherapy Program UPMC Hillman Cancer Center Pittsburgh Pennsylvania USA; ^7^ National Clinical Research Center for Infectious Diseases (SHENZHEN) Shenzhen China

**Keywords:** macrophage, mycobacteria, *Mycobacterium tuberculosis*, necroptosis, *Rhodococcus equi*

## Abstract

Macrophages are the predominant cell type infected by *Mycobacterium tuberculosis* (Mtb) in tuberculosis (TB). Death of Mtb‐infected macrophages promotes tissue pathology and releases Mtb to infect other cells, suggesting that inhibiting the death of Mtb‐infected macrophages could be an adjunctive treatment of TB. Prospects for such an intervention depend on identifying the molecular pathways leading to cell death. We previously reported that the death of Mtb‐infected mouse macrophages *in vitro* depends on type I interferons (IFNs) and lysosomal membrane permeabilization contributes to cell death. Here, we report that the death of Mtb‐infected primary mouse macrophages *in vitro* became necroptotic in the presence of a pan‐caspase inhibitor, benzyloxycarbonyl‐Val‐Ala‐Asp‐fluoromethylketone (z‐VAD‐FMK, hereafter called z‐VAD) acting on a target other than caspase‐8. Macrophages infected with *Mycobacterium kansasii* or *Rhodococcus equi* likewise underwent z‐VAD‐dependent necroptosis. In TB‐resistant C57BL/6 mice, the deficiency of the key necroptosis executioner, mixed lineage kinase domain‐like (MLKL), did not affect bacterial burden or pulmonary pathology. In contrast, in mice lacking the IFN repressor SP140, which express high levels of type I IFNs after Mtb infection and develop necrotic pulmonary lesions, MLKL‐deficiency reduced bacterial burden and pathology after high‐dose infection. This report illustrates that off‐target action(s) of a caspase‐8 inhibitor can switch the cell death pathway to necroptosis in macrophages infected with several Gram‐positive pathogens and highlights that necroptosis can exacerbate TB. Identifying the target of z‐VAD may help to delineate how necrotic lesions form in TB.

## Impact statement

Tuberculosis (TB) remains a major threat to human health, with the emergence of drug resistance necessitating adjunctive host‐directed therapies. *Mycobacterium tuberculosis* (Mtb) infection can kill macrophages, which can promote tissue pathology and allow the released Mtb to infect other cells. Here, we find that a pan‐caspase inhibitor benzyloxycarbonyl‐Val‐Ala‐Asp‐fluoromethylketone (z‐VAD‐FMK) hastened the death of macrophages infected with Mtb, *Mycobacterium kansasii* or *Rhodococcus equi*, shifting the cell death to necroptosis. In mice lacking the type I interferon (IFN) repressor SP140, the deficiency of the key necroptosis executioner, mixed lineage kinase domain‐like (MLKL), resulted in less weight loss, lower Mtb burdens, and mitigation of lung pathology after high‐dose Mtb infection, suggesting that necroptosis can contribute to TB pathogenesis in some circumstances.

## INTRODUCTION

Like many pathogens, *Mycobacterium tuberculosis* (Mtb) manipulates important host cellular processes, including cell death pathways, for its own propagation. Virulent Mtb inhibits apoptosis of macrophages^1^, ^2^, the initial and major cellular niche for Mtb infection and replication. Nonetheless, macrophages that fail to clear Mtb eventually die, releasing bacilli that can infect other cells in the same host or be expelled in aerosols to infect others. In addition, release of cellular content contributes to tissue inflammation.

Necroptosis is a type of programmed necrosis that classically requires receptor‐interacting protein kinase 1 (RIPK1), RIPK3, and mixed lineage kinase domain‐like (MLKL)^3^, ^4^, ^5^, ^6^, ^7^, ^8^, ^9^. Necroptosis has been most thoroughly studied in response to TNFα^10^, ^11^. TNFα signaling usually triggers NF‐κB and mitogen‐activated protein (MAP) kinase pathways that promote cell survival^12^. However, if the activity of cellular inhibitor of apoptosis proteins (cIAPs) is inhibited by the second mitochondria‐derived activator of caspases (Smac/DIABLO) protein or small molecule mimetics of Smac (Smac mimetics), caspases are activated, leading to apoptosis^13^, ^14^, ^15^. If caspases are also inhibited, the death mode shifts to necroptosis, provided that the downstream necroptosis machinery is intact. RIPK1 and RIPK3, interacting through their respective homotypic interaction motifs (RHIMs)^16^, join with MLKL to form the necrosome^8^, ^9^. The interaction between RIPK1 and RIPK3 drives the autophosphorylation of RIPK3, enabling its recruitment and phosphorylation of MLKL^5^, ^8^. Phosphorylated MLKL oligomerizes and forms a pore in phospholipid‐rich cell membranes, resulting in cell death^17^, ^18^. Necroptosis can also ensue in response to Toll‐like receptor (TLR) signaling or viral infection^19^, ^20^. In the latter two cases, the RHIM domain‐containing proteins TIR‐domain‐containing adapter‐inducing interferon‐β (TRIF) and Z‐DNA‐binding protein 1 (ZBP1), respectively, mediate the interaction with RIPK3. It was recently reported that mitochondrial DNA is released into the cytosol upon necroptosis induction and activates the cGAS‐STING pathway to promote type I IFN expression^21^. *In vivo*, necroptosis has been found in disorders and states ranging from nerve injury to senescence of the mouse male reproductive system^22^, ^23^, ^24^, ^25^.

Whether Mtb infection results in necroptosis is inconclusive. In one study, macrophages were protected from Mtb‐induced cell death *in vitro* by depletion of RIPK3 or MLKL, and the numbers of Mtb and neutrophils in the lungs were modestly reduced in *Ripk3* knockout mice compared with wild‐type (WT) mice after high‐dose intravenous infection^26^. However, in other studies, low‐dose aerosol infection led to a similar course of disease in WT, *Mlkl*‐deficient, and *Ripk3*‐deficient C57BL/6 mice^27^, ^28^. Tuberculosis (TB) necrotizing toxin (TNT) is secreted by Mtb into macrophages, where its nicotinamide adenine dinucleotide glycohydrolase activity led to necroptosis that was dependent on RIPK3 and MLKL, but not on TNFα or RIPK1^29^. However, Mtb lacking TNT was not defective for the course of infection in mice^30^. Components of the necroptotic machinery were reported to increase in macrophages after Mtb infection both *in vivo* and *in vitro*, due in part to the type I IFN signaling induced by the infection. However, neither chemical nor genetic perturbation of the necroptosis pathway reduced the death of Mtb‐infected macrophages^27^, ^28^, ^31^. *Mycobacterium marinum* infection of zebrafish or cells of a human macrophage cell line led to a form of necrosis dependent on TNFα and the production of mitochondrial reactive oxygen species (ROS), without relying on RIPK1, RIPK3, or MLKL^32^.

We previously reported that Mtb infection alone did not elicit necroptosis in mouse macrophages *in vitro* ^31^. However, in this study, we found that addition of benzyloxycarbonyl‐Val‐Ala‐Asp‐fluoromethylketone (z‐VAD‐FMK, hereafter called z‐VAD), a presumed pan‐caspase inhibitor, led to rapid cell death of Mtb‐infected macrophages by necroptosis in a manner dependent on type I IFNs, TNFα, RIPK1, RIPK3, and MLKL, but not dependent on inhibition of caspases. The Gram‐positive pathogens *Mycobacterium kansasii* and *Rhodococcus equi* also elicited macrophage necroptosis in the presence of z‐VAD. Finally, we observed that MLKL contributes to TB pathology in high‐dose infection of mice prone to the formation of necrotic tuberculous lesions. The evidence highlights that necroptosis of macrophages infected with various pathogens can be elicited *in vitro* through pharmacologic action on a target(s) other than caspase‐8 and can occur *in vivo*, depending on the genetic background of the host.

## RESULTS

### Mtb infection in the presence of z‐VAD leads to macrophage necroptosis

Bone marrow‐derived macrophages (BMDMs) from C57BL/6 mice died within 24 hours post infection (hpi) with Mtb if they were also exposed to the pan‐caspase inhibitor z‐VAD, a time at which death was not yet evident in macrophages infected with Mtb alone (Figure 1A,B). However, the caspase‐1 inhibitor Ac‐YVAD‐CMK (YVAD), the caspase‐8 inhibitor z‐IETD‐FMK (IETD), the caspase‐3 inhibitor Ac‐DEVD‐CHO, another pan‐caspase inhibitor Q‐VD‐OPH, or even their combinations had no such effect (Figures 1A, S1A and S1B). The RIPK1 inhibitor necrostatin 1 (Nec‐1) and the RIPK3 inhibitor GSK'872 that potently inhibits RIPK3 kinase activity^33^ reverted the death phenotype seen with z‐VAD (Figures 1B and S1A,B) without exerting a toxic effect (Figure S1C). Consistent with the cell death in this setting being necroptotic, Mtb infection combined with z‐VAD treatment triggered time‐dependent phosphorylation of RIPK1 and MLKL (Figure S1D). Nec‐1 inhibited both phosphorylation events, while GSK'872 inhibited phosphorylation of MLKL but not RIPK1 (Figures 1C and S1D). Moreover, a loss‐of‐function mutation in RIPK3 and deficiency of MLKL each conferred resistance to z‐VAD‐dependent, Mtb‐related necroptosis (Figures 1D,E and S1E), as well as to necroptosis in response to the classical necroptosis‐promoting combination of TNFα, Smac‐mimetic birinapant, and z‐VAD (hereafter called TSZ) without Mtb (Figure S1F,G). The combination of TNF‐α, Smac‐mimetic birinapant, and Q‐VD‐OPH (hereafter called TSQ) triggered slower necroptosis than TSZ, but GSK'872 blocked necroptosis in both cases (Figure S1H).

**Figure 1:**
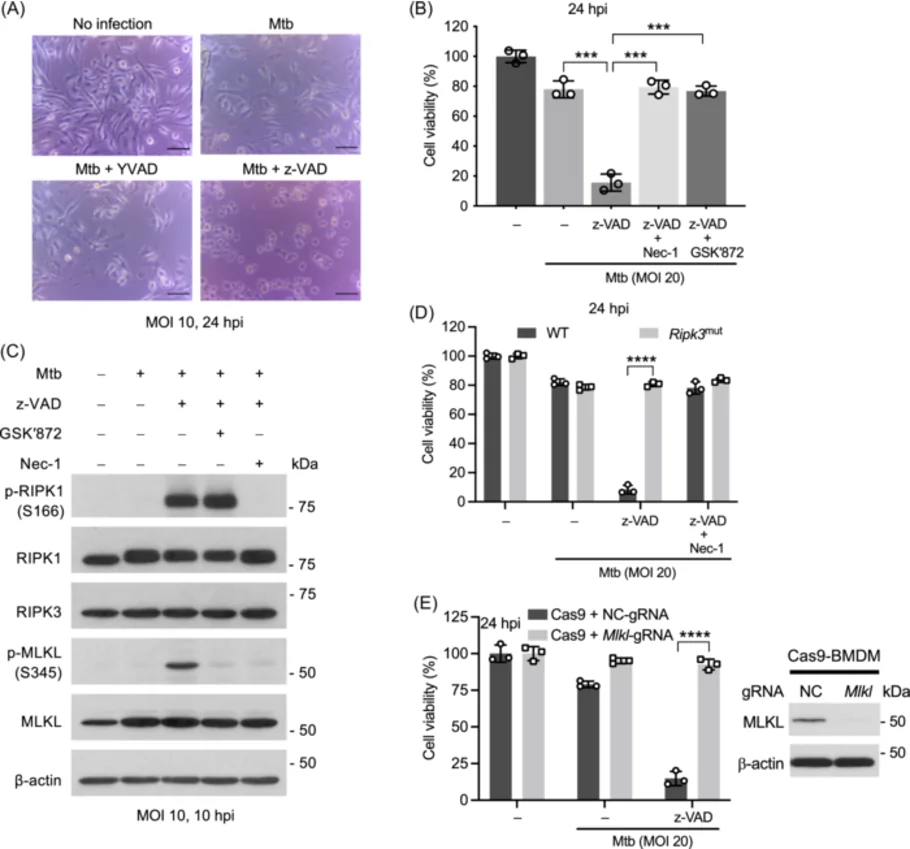
Mtb infection in the presence of z‐VAD treatment induces RIPK1–RIPK3–MLKL‐dependent necroptosis. BMDMs were pretreated with the indicated compounds at the concentration of 10 μM, except 1.5 μM for GSK′872 for 2 h before infection of Mtb and then at the indicated concentration of 20 μM, except 3 μM for GSK′872 after washing Mtb away till harvest. (A) Photomicrographs of cells at 24 hpi. Scale bar, 50 μm. (B) Cell viability measured by the ATP assay at 24 hpi. (C) Effects of RIPK1 and RIPK3 inhibitors (Nec‐1 and GSK′872, respectively) on necroptotic activation of Mtb + z‐VAD‐treated BMDMs. Immunoblots of cell lysates harvested at 10 hpi are shown. (D, E) Effects of RIPK3 (D) and MLKL (E) deficiency on Mtb‐related macrophage necroptosis. Cell viability was measured by the ATP assay at 24 hpi. Data are means ± SD of three technical replicates (B, D, and E) and representative of two experiments. ***p < 0.001; ****p < 0.0001 (two‐tailed unpaired Student's *t* test). BMDMs, bone marrow‐derived macrophages; hpi, hours post infection; MLKL, mixed lineage kinase domain‐like; MOI, multiplicity of infection; Mtb, *Mycobacterium tuberculosis*; NC, negative control; RIPK, receptor‐interacting protein kinase; YVAD, caspase‐1 inhibitor Ac‐YVAD‐CMK; and z‐VAD, benzyloxycarbonyl‐Val‐Ala‐Asp‐fluoromethylketone.

Even though Mtb + z‐VAD robustly induced phosphorylation of mouse MLKL at S345, these stimuli did not induce detectable phosphorylation of mouse RIPK3 at T231 and S232 (Figure S1I). In contrast, both sites were phosphorylated in response to TSZ (Figure S1I). Thus, both Mtb‐related, z‐VAD‐dependent necroptosis and TSZ‐stimulated necroptosis involve RIPK3 and its kinase activity, but lead to differential post translational modification of RIPK3.

### Mtb‐related, z‐VAD‐dependent necroptosis relies on TLR2–TNFα signaling as well as type I IFN signaling induced by infection

Classically, necroptosis involves TNFα signaling. Mtb‐infected macrophages produce TNFα predominantly in response to induction of the TLR2–MYD88–NF‐κB pathway^34^, ^35^, ^36^ and respond to it in autocrine or paracrine fashion through TNF receptor 1 (TNFR1). Consistent with this, we found that BMDMs deficient in TNFα or TNFR1 were resistant to Mtb‐related, z‐VAD‐dependent necroptosis (Figures 2A and S2A,B). Likewise, genetic loss of TLR2, the major TLR for recognizing Mtb, or MYD88, a key adaptor downstream of TLR2, abolished the production of TNFα after Mtb infection (Figures 2B and S2C) and prevented Mtb‐related necroptosis (Figures 2A and S2D,E). In contrast, TLR4, 5, 7, and 9, adaptor proteins TRIF and sterile alpha and TIR motif containing 1 (SARM1) were each dispensable for necroptosis (Figures 2A and S2D–H), and TRIF was dispensable for TNFα production after Mtb infection (Figure 2B).

**Figure 2:**
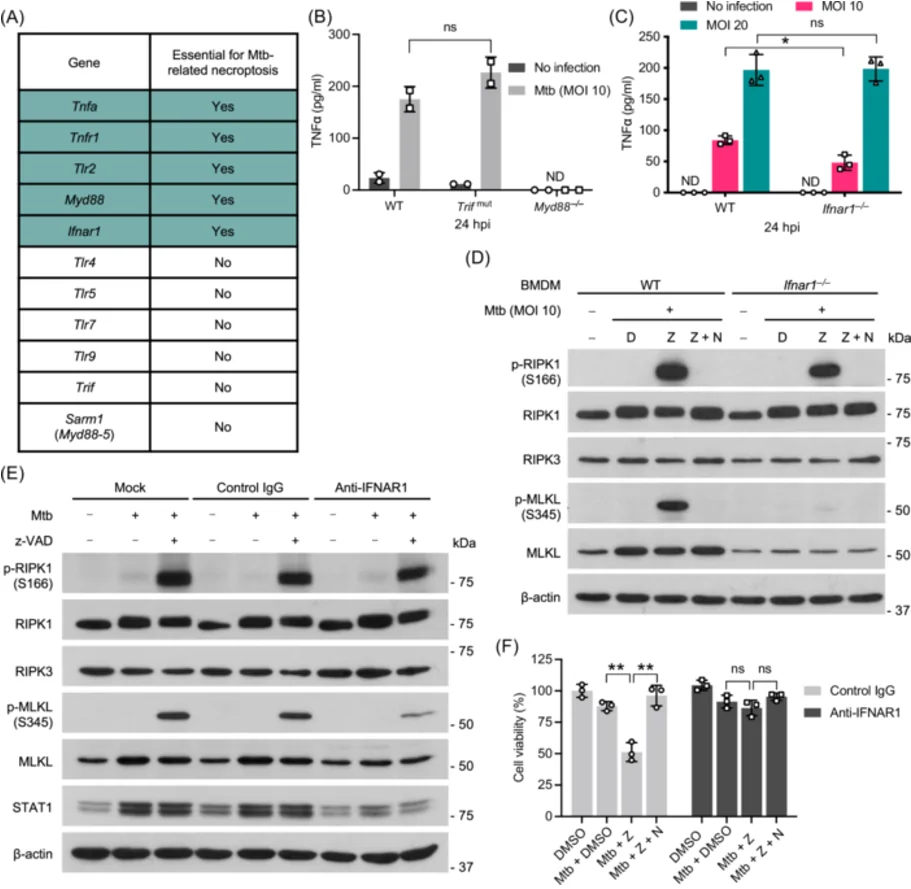
Mtb‐related necroptosis relies on Toll‐like receptor (TLR) – tumor necrosis factor (TNF) signaling as well as type I interferon (IFN) signaling. (A) Effects of various gene deficiencies on Mtb‐related macrophage necroptosis based on the cell viability assay in the presence of Mtb + z‐VAD. BMDMs from wild‐type (WT) and indicated knockout mice were infected with Mtb at an MOI of 20 in the presence of z‐VAD, as in Figure 1, and cell viability was assayed at 24 hpi by measuring the ATP level. Detailed data are shown in Figure S2. (B, C) Effects of genetic deficiency on TNFα secretion by Mtb‐infected macrophages. BMDMs were infected with Mtb at indicated MOI and the cell culture supernatant was harvested at 24 hpi and analyzed by Enzyme‐Linked Immunosorbent Assay (ELISA). (D) Effects of *Ifnar1* deficiency on Mtb‐related necroptosis activation. BMDMs were treated as in (A) and cells were harvested at indicated time for Western blot. (E, F) Effects of blockade of type I IFN signaling on macrophage necroptosis. Immunoblots of cell lysates (E) and cell viability measured by the ATP assay (F) are shown. BMDMs were pretreated with the indicated antibody at 20 μg/ml and compounds of Z or Z and N 2 h before infection and the treatment was continued till harvest at 10 hpi (E) or at 24 hpi (F). Data are means ± SD of two (B) or three technical replicates (C, F) and representative of two experiments. D, DMSO; Z, z‐VAD; N, Nec‐1; ND, not detected; ns, not significant; *p < 0.05; and **p < 0.01 (two‐tailed unpaired Student's *t* test).

Without pathogen infection, in some cell lines, the combination of TNFα and z‐VAD induces necroptosis^33^, ^37^, but this was not the case with macrophages in this study (Figure S3A). Thus, the TNFα produced in response to Mtb is not the only macrophage response to Mtb that is involved in their necroptotic death.

In contrast, type I IFNs, another type of cytokines secreted by Mtb‐infected macrophages, were required for their necroptotic death. The absence of *Ifnar1* did not affect TNFα production (Figure 2C) but made the macrophages resistant to Mtb‐related, z‐VAD‐dependent necroptosis (Figures 2A and S2I). It was recently reported that SP140 represses the expression of type I IFNs through the RESIST (regulated stimulator of interferon via stabilization of transcript) pathway and that *Sp140* ^−/−^ macrophages and mice showed elevated type I IFNs after Mtb infection or treatment by various ligands including TNFα, lipopolysaccharide (LPS), and poly (I:C), making *Sp140* ^−/−^ mice more susceptible to TB^38^, ^39^. Indeed, we found that deficiency of SP140 rendered BMDMs more susceptible to Mtb‐related, z‐VAD‐dependent necroptosis (Figure S3B).

*Ifnar1* ^−/−^ BMDMs showed a similar level of phosphorylation of RIPK1 as WT cells but less phosphorylated MLKL and less total MLKL, because upregulation of MLKL expression was abrogated in *Ifnar1* ^−/−^ BMDMs (Figure 2D), consistent with reports that *Mlkl* is an interferon‐stimulated gene (ISG)^27^, ^40^. Blockade of type I IFN signaling with anti‐IFNAR1 mAb, starting 2 h before infection, prevented the upregulation of MLKL and STAT1, another ISG, seen in WT macrophages, without affecting the basal levels of MLKL and STAT1 (Figure 2E). Anti‐IFNAR1 mAb did not affect RIPK1 phosphorylation, but led to decreased MLKL phosphorylation (Figure 2E) and significant protection from Mtb‐related, z‐VAD‐dependent necroptosis (Figure 2F), suggesting that the acute induction of MLKL by type I IFN signaling contributes to the necroptosis in this setting.

Although Mtb infection of macrophages induced the production of TNFα and type I IFNs, and both were essential for induction of Mtb‐related, z‐VAD‐dependent necroptosis, the combination of TNFα, IFN‐β, and/or IFN‐α as well as z‐VAD did not induce necroptosis of uninfected BMDMs (Figure S3C). Thus, the contribution of Mtb to z‐VAD‐dependent necroptosis of the macrophages in these experiments includes at least one additional effect beyond induction of TNFα and type I IFNs.

### Mtb‐related, z‐VAD‐dependent necroptosis is conserved in a human macrophage cell line

Mtb infection combined with z‐VAD treatment resulted in faster cell death of the human U937 macrophage cell line than Mtb infection alone (Figure 3A) and led to phosphorylation of human MLKL (Figure 3B). Nec‐1 and GSK'872 abolished cell death (Figure 3A) and MLKL phosphorylation (Figure 3B). The human MLKL inhibitor necrosulfonamide (NSA) inhibited cell death but not MLKL phosphorylation (Figure 3A,B). This is consistent with the report that NSA interacts with the MLKL N‐terminal domain without affecting MLKL phosphorylation and this is sufficient to block translocation of phosphorylated MLKL to the membrane^8^, ^18^.

**Figure 3:**
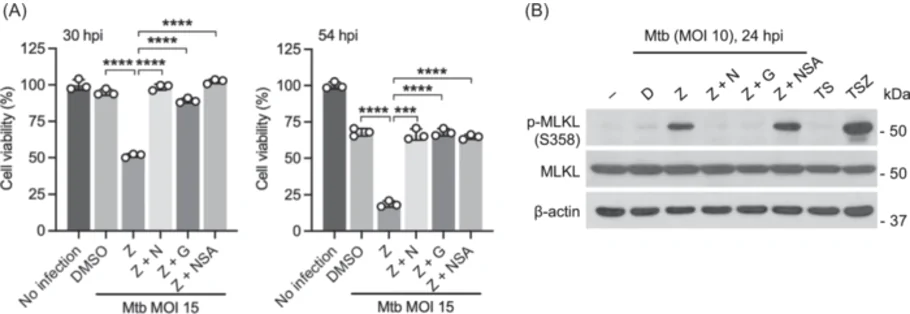
Mtb‐related necroptosis is conserved in human macrophages. Human U937 cells were differentiated with 10 ng/ml phorbol 12‐myristate 13‐acetate (PMA) one day before infection. Two hours before Mtb infection, cells were switched to regular media without PMA and pretreated with indicated compounds at concentration of 10 μM except 1.5 μM for NSA. After infection, cells were washed to remove Mtb and then treated with the compound at the concentration of 20 μM, except 3 μM for NSA till harvest. (A) Effects of necroptosis inhibitors on Mtb + z‐VAD‐induced U937 cell death. Cell viability was measured by the ATP assay at 30 hpi (left) and 54 hpi (right). (B) Effects of necroptosis inhibitors on Mtb + z‐VAD‐triggered MLKL phosphorylation on U937 cells. Immunoblots of cell lysates harvested at 24 hpi are shown. Data are means ± SD of three technical replicates (A) and representative of two experiments. G, GSK'872; NSA, necrosulfonamide; TS, combination of TNFα and Smac mimetic birinapant; TSZ, combination of TNFα, Smac‐mimetic birinapant, and z‐VAD. ***p < 0.001; and ****p < 0.0001 (two‐tailed unpaired Student's *t* test).

### z‐VAD‐dependent necroptosis is also present in macrophages infected with certain other Gram‐positive bacteria

In addition to Mtb, we wondered whether necroptosis also occurred in macrophages infected with other mycobacteria or Gram‐positive bacteria. Infection of BMDMs with *M. kansasii*, the most pathogenic nontuberculous mycobacterium^41^, ^42^, also resulted in robust necroptosis dependent on *Mlkl* in the presence of z‐VAD, but not Q‐VD‐OPH, while *Mycobacterium abscessus* or *M. marinum* combined with z‐VAD was not able to induce necroptosis (Figure 4A). Nec‐1 blocked cell death induced by *M. kansasii* (Figure 4A), further confirming that this cell death is necroptosis and depends on RIPK1.

**Figure 4:**
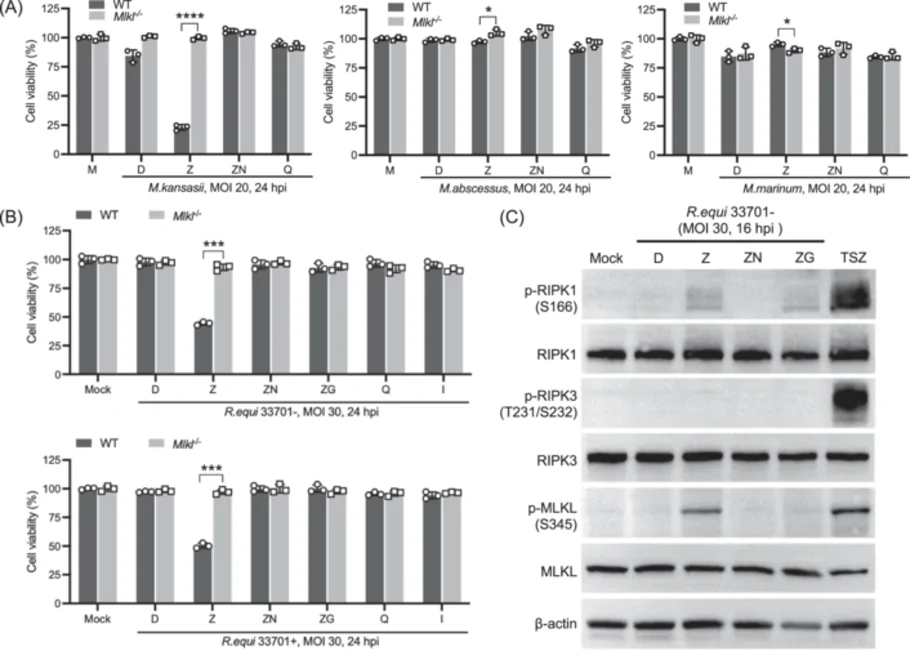
z‐VAD‐dependent necroptosis occurs in macrophages challenged by other Gram‐positive bacteria. Macrophages were pretreated with the indicated compounds at the concentration of 10 μM except 1.5 μM for GSK'872 for 2 h before pathogen infection and then at the concentration of 20 μM, except 3 μM for GSK'872. (A, B) Cell viability of BMDMs measured by the ATP assay after mycobacteria (A) or *Rhodococcus equi* (B) infection. (C) Immunoblots of macrophage cell lysates after *R. equi* infection. J774A.1 cells were harvested at 16 hpi for Western blot. Data are means ± SD of three technical replicates (A and B) and representative of at least two experiments. Q, Q‐VD‐OPH; I, IETD. *p < 0.05; ***p < 0.001; and ****p < 0.0001 (two‐tailed unpaired Student's *t* test).

*R. equi*, a Gram‐positive opportunistic pathogen of humans, is evolutionarily related to Mtb and primarily infects and replicates in alveolar macrophages^43^. It was recently reported that *R. equi* also induces a type I IFN response dependent on the host cGAS‐STING pathway^44^. Indeed, like other mycobacteria, *R. equi* infection of BMDMs elicited upregulated expression of type I IFNs, downstream ISGs, and other inflammatory cytokines including TNFα, independent of its virulence plasmid (Figure S4A). Therefore, we wondered whether *R. equi*‐infected macrophages were predisposed to necroptosis. Like Mtb, *R. equi*‐infected BMDMs underwent fast cell death in the presence of z‐VAD, but not in the presence of Q‐VD‐OPH or IETD, and this cell death could be blocked by Nec‐1 or GSK'872 (Figure 4B). Furthermore, deficiency of MLKL in BMDMs afforded complete protection (Figure 4B). In addition, z‐VAD‐hastened cell death was seen in macrophages infected with either avirulent (33701‐) or virulent (33701+) *R. equi* (Figure 4B), consistent with the expression of type I IFNs, ISGs, and TNFα not relying on its virulence plasmid.

The mouse macrophage cell line J774A.1 underwent *R. equi*‐related, z‐VAD‐dependent necroptosis similarly to BMDMs relying on RIPK3 and MLKL (Figure S4B). *R. equi* infection plus z‐VAD robustly induced phosphorylation of RIPK1 and MLKL (S345), but not phosphorylation of RIPK3 at T231 and S232 in J774A.1 cells (Figure 4C), echoing what we saw in BMDMs infected with Mtb and treated with z‐VAD.

### z‐VAD is very likely to act on non‐caspase‐8 targets in macrophages undergoing Mtb‐ or *R. equi*‐related necroptosis

As germline caspase‐8 knockout in mice results in embryonic lethality and caspase‐8 is essential for the survival of mouse macrophages^45^, ^46^, ^47^, ^48^, we were not able to completely knock out the *Casp8* gene in J774A.1 cells. Instead, we used macrophages haplo‐deficient in *Casp8*, which had drastically reduced expression of caspase‐8 protein (Figures 5A and S5A). The haplo‐deficient cells showed pronounced sensitivity to *R. equi* infection; however, the addition of z‐VAD still resulted in more cell death than in the DMSO control, which could be reverted to a similar level by Nec‐1 and GSK'872 (Figure 5B). These results suggest that inhibition of caspase‐8 is not the mechanism through which z‐VAD mediates necroptosis of *R. equi*‐infected macrophages.

**Figure 5:**
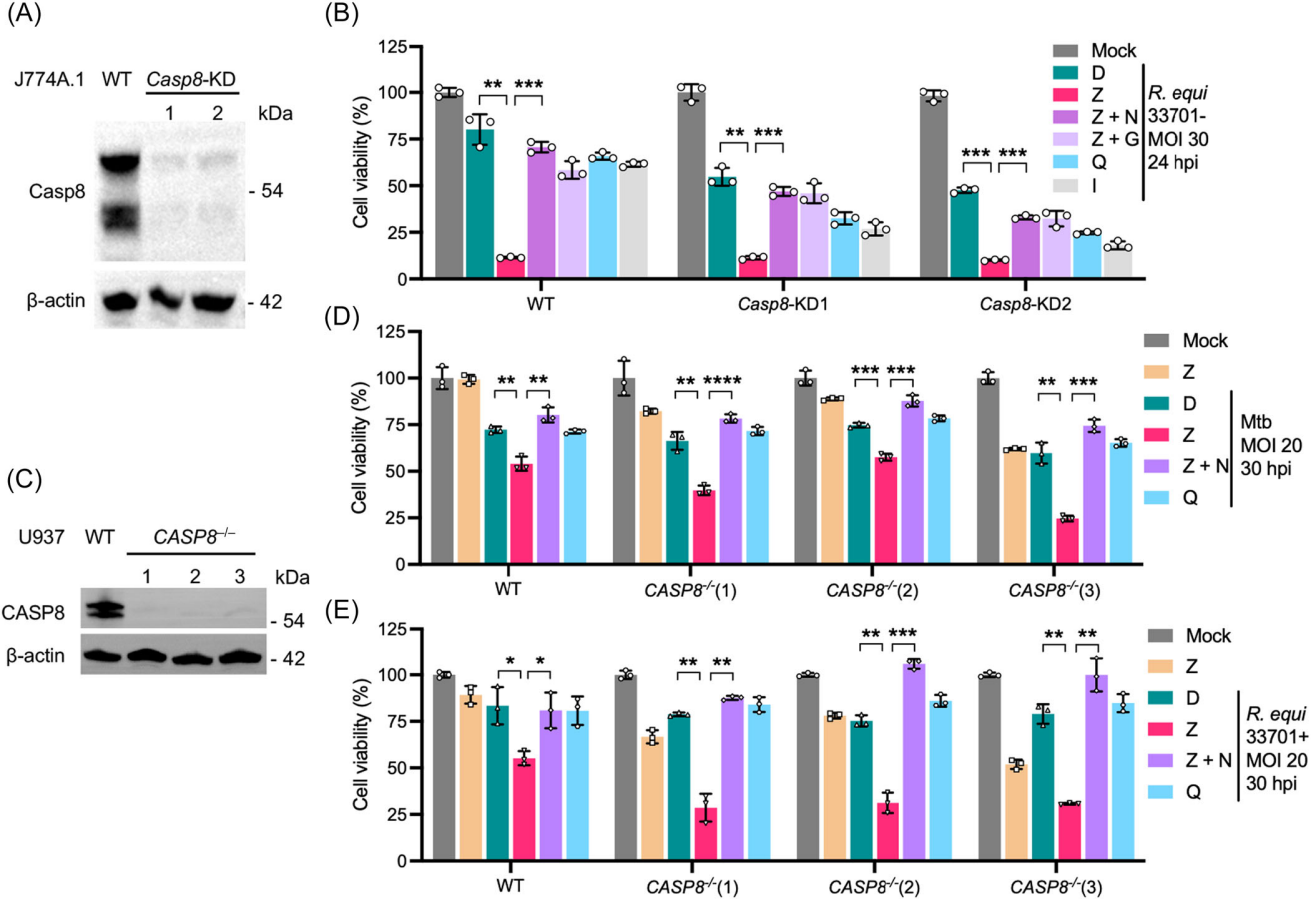
z‐VAD is very likely to act on non‐caspase‐8 targets in macrophages undergoing both Mtb‐ and *R. equi*‐related necroptosis. (A, B) Impact of caspase‐8 knockdown in J774A.1 on *R. equi*‐related necroptosis. Validation of caspase‐8 knockdown efficiency by Western blot (A) and cell viability after *R. equi* infection at an MOI of 30 measured by the ATP assay at 24 hpi (B) are shown. *Casp8*‐KD, caspase‐8 knockdown. (C–E) Impact of caspase‐8 deficiency in U937 on Mtb or *R. equi*‐related necroptosis. Validation of caspase‐8 knockout by Western blot (C) and cell viability after Mtb (D) or *R. equi* (E) infection at the indicated MOI measured by the ATP assay are shown. For infection experiments, cells were pretreated with the indicated compounds at the concentration of 10 μM except 1.5 μM for GSK'872 for 2 h before pathogen infection and then at the concentration of 20 μM except 3 μM for GSK′872. Data are means ± SD of three technical replicates (B, D, and E) and representative of at least two experiments. *p < 0.05; **p < 0.01; ***p < 0.001; and ****p < 0.0001 (two‐tailed unpaired Student's *t* test).

To further delineate the role of caspase‐8, we knocked out both *CASP8* alleles in human U937 macrophages (Figures 5C and S5B). Unlike in mice, where caspase‐8 knockout results in embryonic lethality, homozygous loss of caspase‐8 is compatible with human development, although it leads to immune deficiency and dysregulation^49^, ^50^, ^51^. Similarly, in the context of Mtb or *R. equi* infection, z‐VAD treatment still led to more cell death than in DMSO‐treated controls. This additional cell death could also be reverted by the addition of Nec‐1 (Figure 5D,E), echoing what we observed with *Casp8*‐KD J774A.1 mouse cells.

These data strongly support the fact that z‐VAD is very likely to act on non‐caspase‐8 targets in macrophages undergoing Mtb‐ or *R. equi*‐related necroptosis.

### Necroptosis appears to contribute to disease pathology in *Sp140* ^−/−^ mice infected with a high Mtb burden

We then confirmed and extended the results of Stutz et al.^27^ using *Mlkl* ^+/+^ and *Mlkl* ^−/−^ mice on the C57BL/6 background infected with 200–300 colony‐forming units (CFU) of Mtb by inhalation. Starting from Day 28 post infection, we observed an increase in the MLKL protein level in lung homogenates from *Mlkl* ^+/+^ mice but not from *Mlkl* ^−/−^ mice; yet, we did not detect phosphorylation of MLKL (Figure S6A). Moreover, there was no significant difference between *Mlkl* ^+/+^ and *Mlkl* ^−/−^ mice with respect to the Mtb burden in lungs, spleens, and livers (Figure S6B).

Given that *Mlkl* is an ISG and previous reports showed that *Sp140* ^−/−^ mice had elevated IFN‐I levels after Mtb infection due to abrogation of negative regulation of *Ifnb1* mRNA by SP140^38^, ^39^, we wondered whether the deficiency of SP140 would potentiate the induction of *Mlkl* and provide a more sensitive model for examination of the roles of necroptosis in TB disease progression. We infected *Sp140* ^−/−^ and *Sp140* ^−/−^ *Mlkl* ^−/−^ mice on the C57BL/6 background with 400–500 CFU of Mtb by inhalation. Due to the relatively low virulence of this particular strain, the bacterial burden in lungs of *Sp140* ^−/−^ mice reached only around 10^5^ CFU on Day 58 post infection (Figure S7A). Additional loss of *Mlkl* did not affect Mtb burdens in lungs, spleens, or livers, nor did it result in significant weight change (Figure S7A,B). The ratio of lung weight/whole body weight was lower in Mtb‐infected *Sp140* ^−/−^ *Mlkl* ^−/−^ mice than in Mtb‐infected *Sp140* ^−/−^ mice; yet, the lung pathology and spleen/body weight ratios were similar in both *Sp140* ^−/−^ and *Sp140* ^−/−^ *Mlkl* ^−/−^ mice (Figure S7C–E).

However, when mice were infected with an Mtb inoculum high enough to cause weight loss in *Sp140* ^−/−^ mice, *Mlkl* deficiency in the *Sp140* ^−/−^ background significantly attenuated weight loss and also reduced Mtb burdens in the lungs and spleen (Figure 6A,B), leading to less inflammation in both organs, as judged by organ/whole body weight ratio as well as by hematoxylin and eosin staining of infected lungs (Figure 6C–E). Immunohistochemistry detected MLKL in infected *Sp140* ^−/−^ lungs, but not in uninfected *Sp140* ^−/−^ or infected *Sp140* ^−/−^ *Mlkl* ^−/−^ lungs (Figure S8A). Consistently, we observed the induction of MLKL expression in lung homogenates from Mtb‐infected *Sp140* ^−/−^ mice, but not *Sp140* ^−/−^ *Mlkl* ^−/−^ mice (Figure S8B). However, we could not detect phosphorylation of mouse MLKL at S345 in lung homogenates, possibly due to its low abundance, rapid signal decay, or interference with antibody binding by post‐translational modifications near S345 (Figure S8B). Nevertheless, the impact of *Mlkl* deficiency suggests that necroptosis contributes to TB pathology in TB‐susceptible *Sp140* ^−/−^ mice.

**Figure 6:**
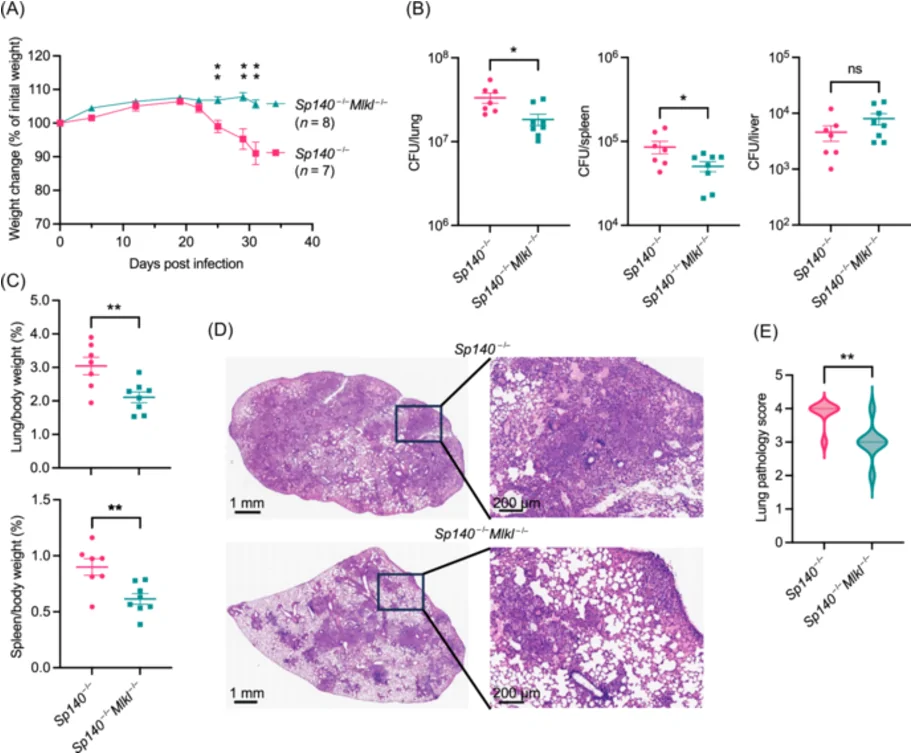
The absence of MLKL delays tuberculosis progression in Mtb‐infected *Sp140* ^*−*/−^ mice. *Sp140* ^*−*/−^ and *Sp140* ^*−*/−^ *Mlkl* ^*−*/−^ mice were infected with 4000–6000 CFU of Mtb on Day 0. (A) Weight changes of Mtb‐infected mice from Day 0 to 31. (B) Mtb burdens in the lungs (left), spleens (middle), and liver (right) of mice. (C) Ratio of lung (top) or spleen (bottom) weight to whole body weight in Mtb‐infected mice. (D) Representative hematoxylin and eosin staining of lung sections of Mtb‐infected mice. (E) Pathology scores of lung sections of Mtb‐infected mice. For (B–E), mice were euthanized on Day 31. *n* = 7 for *Sp140* ^*−*/−^ mice and *n* = 8 for *Sp140* ^*−*/−^ *Mlkl* ^*−*/−^ mice. Data are means ± SEM (A–C). ns, not significant; *p < 0.05; and **p < 0.01 (two‐tailed unpaired Student's *t* test).

## DISCUSSION

Mtb infection, in concert with the type I IFNs that the infection induces, leads to the death of mouse macrophages, at least in part through the impact of host *cis*‐aconitate decarboxylase (ACOD1) on lysosomal membrane permeabilization^31^, ^52^. This study established that the slow macrophage death triggered by Mtb could be rapidly switched to necroptosis in the presence of z‐VAD, while remaining dependent on type I IFNs and TNFα induced by the infection as well as at least one more factor contributed by Mtb or the macrophage response to Mtb. This suggests that after Mtb infection of macrophages, there exists a point of control over the cells' fate. Further study of z‐VAD's targets could help to identify or characterize the critical control point.

What we observed is not restricted to infection of macrophages by Mtb. Infection of mouse BMDMs by *Escherichia coli* strain LF82 or *Citrobacter rodentium* in the presence of z‐VAD induced a form of cell death that could be blocked by Nec‐1, indicating that it is likely necroptosis^53^. Furthermore, this type of cell death relied on both TNF signaling and type I IFN signaling, similar to the necroptosis triggered by Mtb infection in macrophages exposed to z‐VAD. We further identified that some Gram‐positive pathogens including *M. kansasii* and *R. equi* also triggered macrophage necroptosis in the presence of z‐VAD.

The canonical function of z‐VAD is to inhibit caspases in a cell; its ability to promote necroptosis has been ascribed to its inhibition of caspase‐8. Many bacteria and viruses are equipped with machinery to prevent caspase activation^54^, ^55^, ^56^, ^57^, ^58^, ^59^, ^60^. Concurrent infection of macrophages with Mtb and other bacteria or viruses could theoretically lead to necroptosis through caspase inhibition. However, in our studies, caspase inhibition was insufficient to switch pathogen‐infected macrophages to a necroptotic death pathway, based on the following observations: (1) We previously found that Mtb infection alone strongly inhibited the activity of caspase‐8 and caspase‐3 and did not lead to necroptosis^31^. (2) In contrast to z‐VAD, the more specific caspase‐8 inhibitor IETD and the more specific and potent pan‐caspase inhibitor Q‐VD‐OPH did not sensitize pathogen‐infected macrophages to necroptosis. (3) TSZ triggered much faster necroptosis in macrophages than TSQ. (4) z‐VAD is known to inhibit other proteases besides caspases^61^, ^62^, ^63^, ^64^. (5) z‐VAD still triggered fast necroptosis in *Casp8*‐KD J774A.1 and *CASP8* ^−/−^ U937 macrophages upon Mtb/*R. equi* infection. We speculate that z‐VAD may target protein(s) that are expressed in macrophages, but not in the cell lines often used for studies of necroptosis. Cell type‐specific necroptotic signaling has been observed in the differential sensitivity to TNFα on the part of FADD‐deficient MEF cells and Jurkat cells^5^.

Due to the broad‐spectrum inhibition of z‐VAD on other proteases, identifying the critical target(s) for necroptosis in the current setting remains challenging. Traditional affinity‐based enrichment and subsequent mass spectrometric analysis require addition of a linker like biotin or a photoactivatable tag for covalent target trapping^65^, which becomes helpful only if the modified compound remains active on the same target. Cellular thermal shift assays (CETSAs) of whole cells or their lysates combined with highly sensitive mass spectrometric analysis can identify proteins that are bound by a small‐molecule ligand like z‐VAD, providing a powerful tool^65^, ^66^. Whole‐genome‐based or more focused CRISPR‐based genetic screens may also help to pinpoint targets and pathways involved in z‐VAD‐dependent death^67^, ^68^, ^69^. A multi‐pronged strategy may be needed to pinpoint the exact target(s) of z‐VAD in Mtb‐related necroptosis.

Our studies also highlighted the potential of RIPK3 to undergo different patterns of phosphorylation or other post‐translational modification to sustain necroptosis. Many of the serine and threonine residues of RIPK3 are subject to phosphorylation^5^, ^70^. Among them, phosphorylation of human RIPK3 on S227 and murine RIPK3 (mRIPK3) on T231 and S232 is thought to be essential for binding MLKL in a species‐specific way to mediate necroptosis induced by TNF signaling and by LPS + z‐VAD^8^, ^70^, ^71^. Surprisingly, we found that Mtb/*R. equi*‐related, z‐VAD‐dependent necroptosis did not lead to detectable phosphorylation of mRIPK3 on T231 and S232. Nonetheless, necroptosis depended on the presence of RIPK3, was blocked by the RIPK3 inhibitor GSK'872, and was accompanied by phosphorylation of the RIPK3 substrate mMLKL on S345, which in turn was blocked by the RIPK3 inhibitor GSK'872. These results strongly suggest that RIPK3 was activated, but most likely accompanied by phosphorylation on residues other than T231 and S232 or by other types of post‐translational modification to recruit and phosphorylate MLKL. This deviation from the classical paradigm for necroptosis suggests that the pathway is more plastic than had been appreciated.

Studies on necroptosis induced by LPS plus z‐VAD or classical TSZ stimuli suggested that subtle changes in MLKL protein levels can dramatically affect cell sensitivity to necroptotic stimuli and that constitutive IFN signaling maintains cytosolic MLKL above a certain threshold to license necroptosis^72^. In addition to this, we found that the role of type I IFN signaling in the Mtb‐related, z‐VAD‐dependent necroptosis of macrophages includes an acute increase in the expression of MLKL upon Mtb infection.

Results of our experiments with Mtb infection of *Mlkl* ^−/−^ mice are in agreement with previous studies showing that MLKL‐dependent necroptosis is unlikely to account for pathology in Mtb‐infected WT C57BL/6 mice^27^, ^28^. However, WT C57BL/6 mice rarely develop necrotic pulmonary lesions after Mtb infection^73^, which might obscure the possibility of identifying the presence of necroptosis in affected lungs. Deficiency of SP140 in C57BL/6 mice results in a higher type I IFN level and phenocopies the response of 129S2 and C3HeB/FeJ strains to Mtb infection with respect to the formation of necrotic granulomas, as seen in some forms of TB in humans^73^, ^74^. Indeed, compared to *Sp140* ^−/−^ mice, *Sp140* ^−/−^ *Mlkl* ^−/−^ mice showed less weight loss, lower Mtb burdens, and alleviated lung pathology after high‐dose Mtb infection, possibly due to less release of inflammatory material from dying cells. Taken together, our findings suggest that necroptosis can contribute to TB disease under some circumstances. Identification of the relevant targets of z‐VAD may provide more insights into how necroptosis is regulated during TB.

## MATERIALS AND METHODS

### Cell lines and primary macrophages

Human U937 cells (ATCC, Cat# CRL‐1593.2) were cultured in RPMI‐1640 supplemented with 10% fetal bovine serum (FBS), 2 mM l‐glutamine, 10 mM HEPES, pH 7.5, and 1 mM sodium pyruvate, and differentiated with 10 ng/ml phorbol 12‐myristate 13‐acetate (PMA) one day before infection. L929 (ATCC, Cat# CCL‐1) and J774A.1 (ATCC, Cat# TIB‐67) were cultured in Dulbecco's modified Eagle's medium (DMEM) supplemented with 10% FBS, 2 mM l‐glutamine, 10 mM HEPES, pH 7.5, and 1 mM sodium pyruvate. L929‐conditioned medium (LCM) and BMDMs were prepared as previously described^31^. All cells were cultured at 37°C in a 5% CO_2_ incubator unless otherwise specified.

### Mice

WT C57BL/6 (#000664), WT C57BL/6N (#005304), *Ripk3* ^mut^ (#025738), *TNF‐α* ^−/−^ (#005540), *Tnfr1* ^−/−^ (#003242), *Tlr2* ^−/−^ (#004650), *Trif* ^mut^ (#005037), *Tlr5* ^−/−^ (#008377), *Tlr7* ^−/−^ (#008380), *Tlr9* ^−/−^ (#34329‐JAX), *Sarm1* ^−/−^ (#018069), and Cas9 knockin mice (#026179) (all on the C57BL/6 background) were purchased from Jackson Laboratory. *Myd88* ^−/−^ and *Tlr4* ^−/−^ mice were kindly provided by E. Pamer (then at Memorial Sloan Kettering Cancer Center); *Ifnar1* ^−/−^ mice were from J. Sun (Memorial Sloan Kettering Cancer Center); *Mlkl* ^−/−^ mice were from A. Choi (Weill Cornell Medicine); and *Sp140* ^−/−^ mice were from R. Vance (University of California, Berkeley). Cas9‐het mice were prepared and used as previously described^31^. *Mlkl* ^+/+^ and *Mlkl* ^−/−^ mice were bred in‐house from a heterozygote X heterozygote mating scheme and genotyped before infection. *Sp140* ^−/−^ *Mlkl* ^−/−^ mice were generated by regular cross. Mice of both sexes aged 6–12 weeks were used for Mtb infection experiments. At most time points, the mice harvested from different experimental groups were sex‐ and age‐matched. All mice were housed in a specific pathogen‐free facility or a BSL3 vivarium if infected by Mtb. All mouse experiments were approved by and performed in accordance with requirements of the Institutional Animal Care and Use Committees at Weill Cornell Medicine or Southern University of Science and Technology.

### Reagents

Mouse TNF‐α (#T0157) and Nec‐1 (#N9037) were purchased from Sigma. Mouse IFN‐αA (#12100‐1) and mouse IFN‐β (#12405‐1) were from PBL Assay Science. z‐VAD‐FMK (#A1902) was from Apexbio. Smac mimetic birinapant (#501015095), GSK'872 (#5303890001), and z‐IETD‐FMK (#FMK007) were from Fisher Scientific. NSA (#20844), Q‐VD‐OPH (#15260), and Ac‐YVAD‐CMK (#10014) were from Cayman Chemical. Ac‐DEVD‐CHO (#TQ0146) was from TargetMol. Mouse monoclonal anti‐human IFN‐γ Rα chain (as control IgG; #G737) and mouse monoclonal anti‐mouse IFNAR1 (MAR1‐5A3; #I‐1188) were purchased from Leinco Technologies. Antibodies for β‐actin (#sc‐47778) and RIPK3 (#sc‐374639) were from Santa Cruz Biotechnology. The RIPK3 antibody (#2283) was from ProSci. Antibodies for phospho‐RIP1 (Ser166; #31122S), MLKL (#37705S for mouse, #14993S for human), phospho‐MLKL (Ser345; #37333S), and STAT1 (#9172S) were from Cell Signaling Technology. The caspase‐8 antibody (#13423‐1‐AP) was purchased from Proteintech. The phospho‐RIPK3 (Thr231/Ser232; #ab222320) antibody was from Abcam and the RIPK1 antibody (#610459) was from BD Biosciences. Anti‐GAPDH (#G041) was purchased from Abm.

### Bacterial culture and macrophage infection

Mycobacteria were grown in Middlebrook 7H9 medium supplemented with 0.5% glycerol, 10% oleic acid–dextrose–catalase (BD Biosciences; #212351), and 0.02% tyloxapol (7H9 complete medium). Mtb H37Rv, *M. kansasii*, and *M. marinum* M strain were cultured in a 5% CO_2_ incubator at either 37°C (Mtb and *M. kansasii)* or 32°C (*M. marinum*). *M. abscessus* was grown with constant shaking at 37°C. Mycobacterial single cells were prepared as previously described^31^ and added to macrophages to initiate infection. After infection for 4 h (Mtb), 3 h (*M. kansasii*), or 1 h (*M. marinum* and *M. abscessus*), macrophages were washed twice with warm PBS and then fresh medium was replaced. No antibiotics were used in the preparation, infection, or post‐infection incubation of macrophages.

For *R. equi* infection, one colony of *R. equi* was inoculated into 5 ml of brain–heart infusion broth (Thermo Scientific Oxoid; CM1135) and shaken overnight at 30°C, and then, the indicated culture was subcultured into fresh BHI and shaken at 37°C for 3–4 h until the optical density at 600 nm (OD_600_) reached 0.3–0.5. The bacteria were washed twice with PBS and resuspended in DMEM complete medium, and then added to macrophages. One hour later, macrophages were washed twice with warm PBS and medium containing 100 μg/ml gentamicin was replaced to kill extracellular *R. equi*. One hour later, fresh medium containing 10 μg/ml gentamicin was replaced after macrophages were washed with warm PBS.

For Western blot, macrophages were harvested at the indicated time point and lysed with 1X SDS loading buffer at 95°C for 30 min. For quantitative PCR, total RNA was extracted using the EZ‐press RNA Purification Kit (EZBioscience; B0004D). cDNA was synthesized with the Hifair® III 1st Strand cDNA Synthesis Kit (YEASEN; 11141ES10) according to the manufacturers' instructions. qRT‐PCR was performed using ChamQ Universal SYBR qPCR Master Mix (Vazyme; Q711). Data were analyzed on the CFX96 Touch Real‐Time PCR Detection System (Bio‐Rad).

### Cell viability assays

The viability of macrophages was assessed using the CellTiter‐Glo Luminescent Cell Viability Assay (Promega), which measures intracellular ATP levels, following the manufacturer's instructions. Percentage viability was normalized to that of uninfected cells.

### ELISA

Cell culture supernatants after macrophage infection were centrifuged at 21,130*g* for 5 min at 4°C, followed by filtration through a 0.22 μm cellulose acetate membrane before removal from the BSL‐3 facility. Samples were diluted 3‐ to 4‐fold before ELISA was performed in accordance with the manufacturers' instructions.

### Aerosol infection of mice and organ harvest

Mtb infection of mice was carried out as previously described^31^. Briefly, log‐phase cultures of the Mtb H37Rv strain grown in Middlebrook 7H9 medium containing 0.5% glycerol, 10% albumin–dextrose–saline (ADN) supplement, and 0.05% Tween‐80 were used to prepare a suspension enriched in single cells. The required volume was centrifuged at 3082*g* for 10 min, and the resulting pellet was resuspended in PBS. Mice were infected using a Glas‐Col Inhalation Exposure System using the Mtb inoculum prepared as above. On Day 1 post infection, 2 mice were euthanized and lung homogenates in PBS were plated on 7H10 agar supplemented with 0.5% glycerol and 10% OADC to determine the initial bacterial load (detailed infection doses for each experiment are specified in the corresponding figure legends). On indicated days, at least 5 mice were euthanized with CO_2_ and the lungs, livers, and spleens were harvested. Each organ (except for the upper lobe of the left lung) was homogenized in PBS (if the homogenates were to be used for Western blot, the PBS was supplemented with 1% Roche EDTA‐free protease inhibitor cocktail and PhosStop), serially diluted, and plated on 7H10 agar for the determination of CFU at 3 weeks after plating. The upper lobes of the left lungs were fixed in 10% formalin.

For Western blot, lung homogenates were centrifuged at 21,130*g* at 4°C for 20 min. The supernatant was centrifuged again and passed through a 0.22 μm cellulose acetate filter before being removed from the BSL3 facility. The protein concentration was quantified by a BIO‐RAD *DC*™ Protein Assay kit and ~50–60 μg of protein was used for SDS‐PAGE and western blot.

For histopathological analysis, formalin‐fixed lungs were embedded in paraffin, sectioned, and then stained with Hematoxylin and eosin. For immunohistochemical analysis, the sections were incubated with 3% H_2_O_2_ to quench endogenous peroxidase activity, followed by blocking with 5% BSA to reduce nonspecific binding. The sections were then incubated overnight at 4°C with a primary antibody against MLKL (Cell Signaling Technology; 37705S; 1:200), and subsequently with the HRP‐conjugated goat anti‐rabbit IgG secondary antibody for 30 min at room temperature. Immunoreactivity was visualized using diaminobenzidine (DAB). The stained sections were scanned by a digital pathology scanner (KFBIO; KF‐PRO‐120), and pathological severity was scored in a blinded fashion based on the extent of inflammation: 0, 1, 2, 3, and 4 correspond to 0%–4%, 5%–20%, 21%–40%, 41%–60%, and 61%–100% of the lung area affected by inflammation, respectively.

### Generation of J774A.1 and U937 knockout cells

All J774A.1 or U937 knockout cell lines were generated as previously described^75^. 5 × 10^5^ J774A.1 or U937 cells were spin‐infected (800 rpm, 1.5 h, 37°C) with the guide RNA (gRNA) lentivirus packaged from LentiCRISPRv2 vectors. 24 h later, fresh medium was replaced and cells were cultured for 24 h before adding 3 μg/ml puromycin for 3 days. Then, genomic DNA from live cells was prepared, and gRNA efficiency was validated by the SURVEYOR assay. Single cells were then sorted using BD FACS Aria SORP, and the genotype of each cell clone was verified by sequencing of the PCR fragments. The efficiency of specific gene knockout was validated by Western blot. The gRNA sequences are as follows: *Casp8*: 5′‐TGAGATCCCCAAATGTAAGC‐3′; *Ripk3*‐gRNA1: 5′‐GTGGGACTTCGTGTCCGGGC‐3′; *Ripk3*‐gRNA2: 5′‐AACCCGAGTGCCCTCGGCCC‐3′; *Mlkl*‐gRNA1: 5′‐GCGTCTAGGAAACCGTGTGC‐3′; *Mlkl*‐gRNA2: 5′‐TGGGCCGTTTTGATGAAGTC‐3′; and *CASP8*: 5′‐GTGTGGGGTAATGACAATCT‐3′ (for U937).

### Electroporation of gRNAs into Cas9‐expressing BMDMs

LentiGuide plasmids (Addgene, #52963) with the correct gRNA insertion were used as a template to amplify hU6 promoter–gRNA fragments and then electroporated into Cas9‐expressing BMDMs as previously described^31^. The gRNA sequences used are as follows: negative control; 5′‐GAACTCGTTAGGCCGTGAAG‐3′;^75^ *Mlkl*: 5′‐GCACACGGTTTCCTAGACGC‐3′.

### Statistical analyses

Results of cell viability assays and ELISAs in macrophages are represented as means ± SD and analyzed by a two‐tailed unpaired Student's *t* test using GraphPad Prism (Version 10.3.1). Mtb burden in mouse organs is represented as means ± SEM. ns, not significant; *p < 0.05; **p < 0.01; ***p < 0.001; ****p < 0.0001.

## AUTHOR CONTRIBUTIONS

**Rong Hu**: Data curation; formal analysis; investigation; methodology; visualization; writing—original draft. **Pei Li**: Investigation. **Jie Han**: Investigation. **Jiamin Huang**: Methodology. **Guoyi Huang**: Methodology. **Xiuju Jiang**: Investigation. **Daniel Pfau**: Investigation. **Guobao Li**: Methodology. **Yi‐Nan Gong**: Funding acquisition; investigation. **Carl F. Nathan**: Funding acquisition; supervision; writing—review and editing. **Li Zhang**: Conceptualization; formal analysis; funding acquisition; investigation; project administration; supervision; writing—original draft; writing—review and editing.

## ETHICS STATEMENT

All animal experiments were conducted in strict accordance with institutional and national guidelines for the care and use of laboratory animals. The study protocols were reviewed and approved by the Institutional Animal Care and Use Committee (IACUC) of Weill Cornell Medicine (Approval No. 2017‐0019) and Southern University of Science and Technology (Approval No. SUSTech‐JY202503215). All efforts were made to minimize animal suffering and any animal showing severe illness or pain was humanely euthanized in accordance with approved protocols. All procedures involving Mtb were performed in biosafety level 3 (BSL‐3) facilities that are fully compliant with relevant biosafety regulations and guidelines. The Weill Cornell Medicine Institutional Review Board ruled in advance that our use of sections from de‐identified human lungs did not constitute human research, so that IRB approval was not required.

## CONFLICT OF INTERESTS

The authors declare no conflict of interests.

## Supporting information

supporting information‐necroptosis figures‐040726.

## Data Availability

All the data supporting the findings of this study are available within the article and the Supporting Information, or from the corresponding author upon reasonable request.
