# Supplementary material for: z‐VAD‐FMK triggers macrophage necroptosis during mycobacterial infection via a non‐canonical off‐target mechanism
Source: mLife. 2026 Oct 1;5(5):637–50. doi: 10.1002/mlf2.70101 (PMC13628176; doi:10.1002/mlf2.70101)
Supplement: Supplementary file 1 [file MLF2-5-637-s001.pdf]

Figure S1

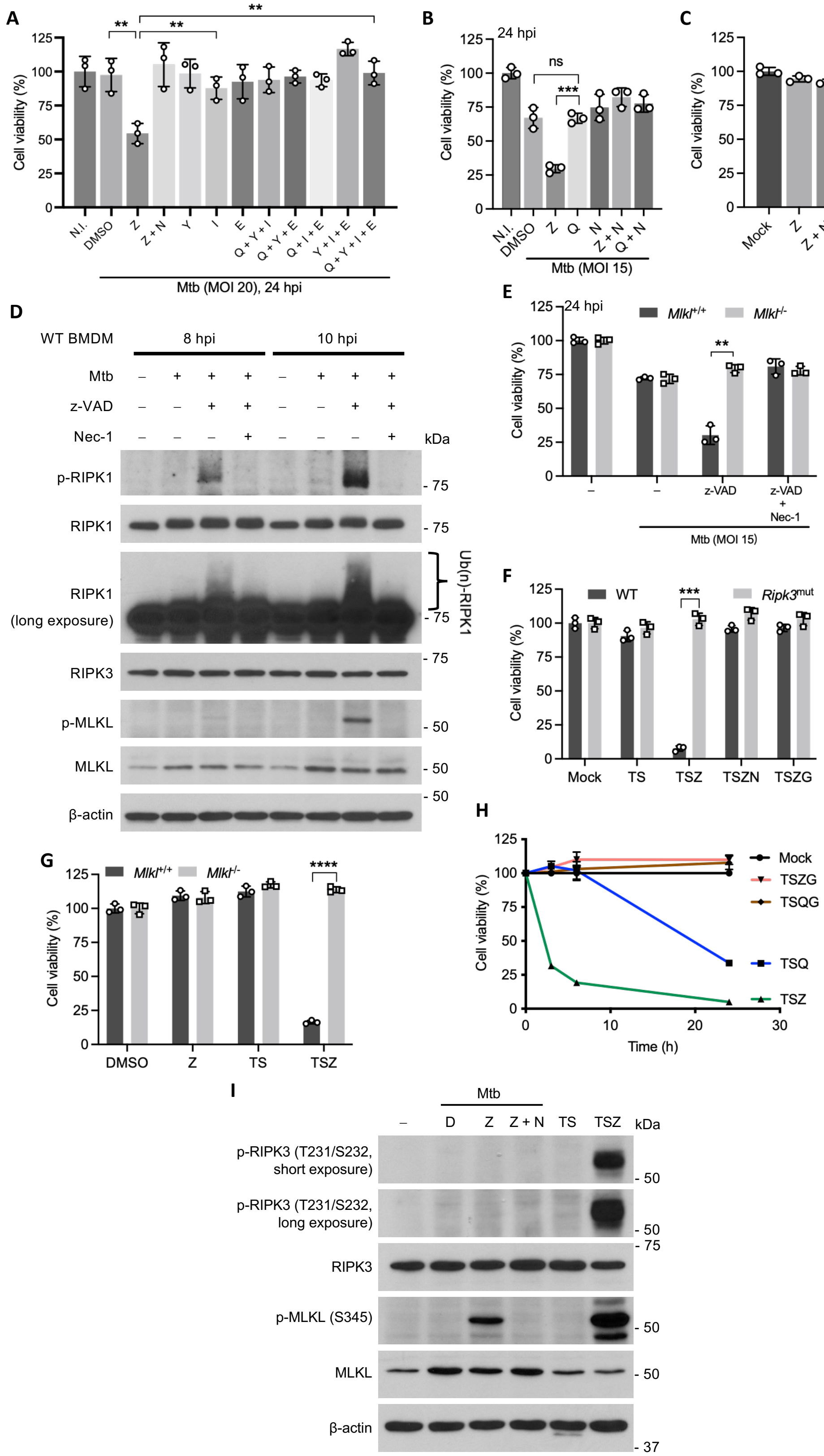

A
B
C
D
E
F
G
H
I
Cell viability (%)
N.I.
DMSO
Z
Z + N
Y
I
E
Q + Y + I
Q + Y + E
Q + I + E
Y + I + E
Q + Y + I + E
Mtb (MOI 20), 24 hpi
24 hpi
ns
Q
N
Q + N
Mtb (MOI 15)
Mock
Z + G
G
WT BMDM
8 hpi
10 hpi
Mtb
z-VAD
Nec-1
kDa
p-RIPK1
RIPK1
RIPK1
(long exposure)
Ub(n)-RIPK1
RIPK3
p-MLKL
MLKL
β-actin
Mlkl+/+
Mlkl-/-
z-VAD + Nec-1
WT
Ripk3mut
TS
TSZ
TSZN
TSZG
TSZG
TSQG
TSQ
Time (h)
D
p-RIPK3 (T231/S232, short exposure)
p-RIPK3 (T231/S232, long exposure)
p-MLKL (S345)

Figure S1. z-VAD sensitizes Mtb-infected BMDMs to necroptosis, which relies on host RIPK3 and MLKL, related to Figure 1. For infection experiment, BMDMs were pretreated with the indicated compounds at concentration of 10 μM, except 1.5 μM for GSK′872 for 2 h before infection of Mtb, and then at concentration of 20 μM, except 3 μM for GSK′872 after washing Mtb away till harvest. (A and B) Effects of various caspase inhibitors on Mtb-infected macrophages. WT BMDMs were pretreated with indicated compounds and cell viability was measured at 24 hpi. (C) z-VAD, Nec-1 and GSK′872 are not cytotoxic without Mtb infection. WT BMDMs were treated with indicated compounds for 24 h and then assayed for cell viability. (D) Mtb + z-VAD induced RIPK1 and MLKL phosphorylation in a time-dependent manner. WT BMDMs were pretreated with indicated compounds 2 h before being infected with Mtb at an MOI of 10. Cell lysates were harvested at indicated time points and subjected to immunoblots. (E) MLKL is essential for Mtb + z-VAD-triggered necroptosis. BMDMs were pretreated with indicated compounds 2 h before being infected with Mtb at an MOI of 15. Cells were assayed 24 hpi. (F and G) Mutation of Ripk3 or loss of Mlkl in BMDMs renders cells resistant to TSZ-triggered necroptosis. BMDMs were treated with indicated compounds for 4 h and then assayed for cell viability by measuring ATP level. (H) The viability curve of WT BMDMs in response to different necroptotic stimulus. Cell viability was measured by ATP level on 3, 6 and 24 h after treatment. (I) Mtb + z-VAD does not induce classical RIPK3 phosphorylation. Cells were treated, infected and assayed as in (D). TS and TSZ treatment lasted for 2 h. N.I., no infection; hpi, hours post infection; MOI, multiplicity of infection; T, TNFα, 10 ng/ml; S, Smac-mimetic birinapant, 10 μM; Z, z-VAD, 20 μM; Q, Q-VD-OPH, 20 μM; G, GSK′872, 3 μM; N, Nec-1, 20 μM; Y, Ac-YVAD-CMK, 20 μM; I, z-IETD-FMK, 20 μM; E, Ac-DEVD-CHO, 20 μM;. Data are shown as means ± SD of three technical replicates (A-C and E-H) and representative of two independent experiments. ns, not significant; ** $p < 0.01$; *** $p < 0.001$ and **** $p < 0.0001$ (two-tailed unpaired Student’s *t* test).

Figure S2

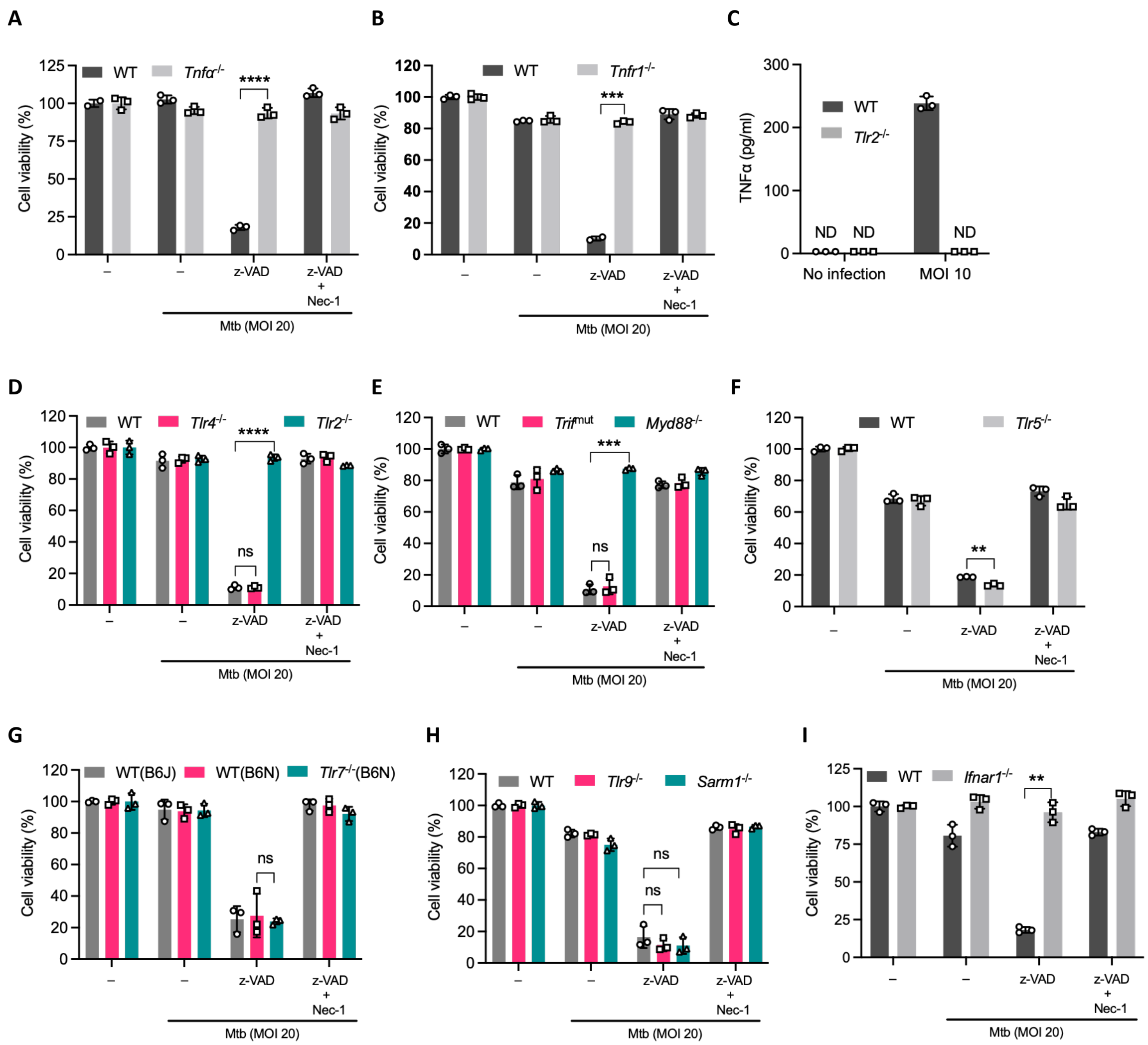

Figure S2. Mtb-related necroptosis depends on TLR2-TNF signaling as well as type I IFN signaling, related to Figure 2. (A–B, D–I) Effects of various gene deficiencies on Mtb-related macrophage necroptosis based on cell viability assay in the presence of Mtb + z-VAD. BMDMs from WT and indicated knockout mice were pretreated with 10 μM indicated compounds for 2 h before infection of Mtb at an MOI of 20 and then at the concentration of 20 μM after washing Mtb away till harvest. Cell viability was assayed 24 hpi by measuring ATP level. Data are summarized in Figure 2A. (C) WT and *Tlr2*$^{-/-}$ BMDMs were infected with Mtb and culture supernatant was harvested 24 hpi and measured by TNFα ELISA. Data are means ± SD of three technical replicates. ND, not detected; ns, not significant; ** $p < 0.01$; *** $p < 0.001$; **** $p < 0.0001$ (two-tailed unpaired Student's *t* test).

Figure S3

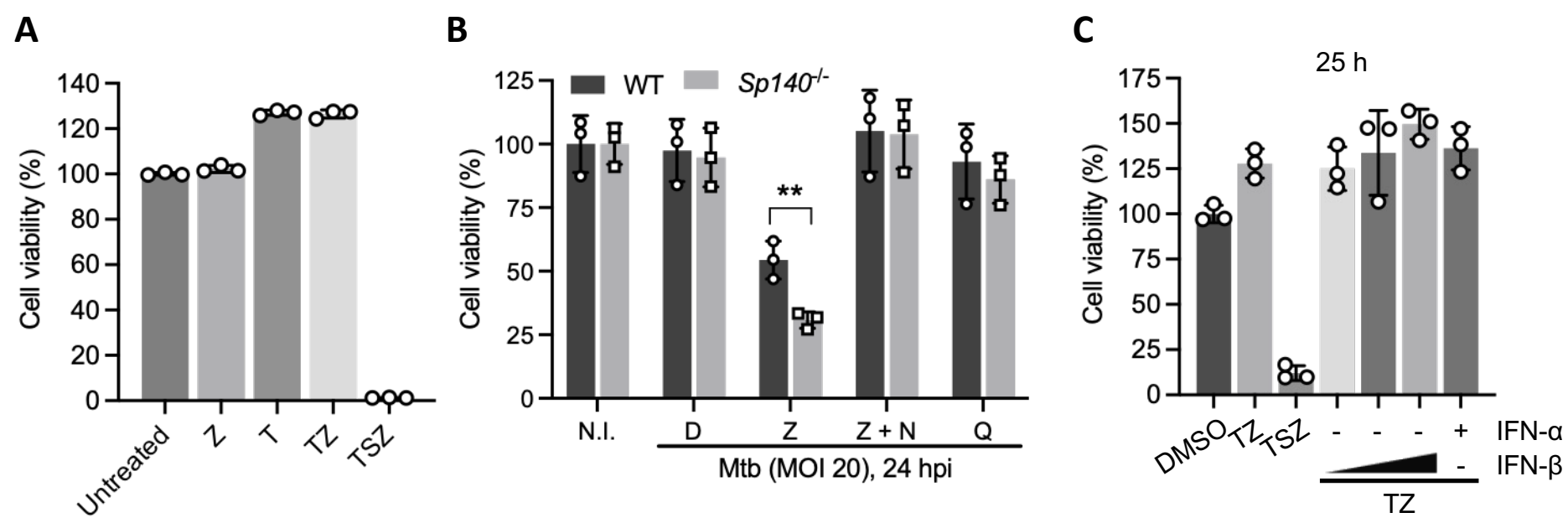

Figure S3. Mtb-induced production of TNFα and type I IFNs are important for necroptosis triggered by Mtb + z-VAD, related to Figure 2. (A) TNFα plus z-VAD does not elicit necroptosis. WT BMDMs were treated with indicated compounds for 31 h and cell viability was analyzed by measuring ATP level. (B) Effects of Sp140 deficiency on Mtb-related macrophage necroptosis. BMDMs were pretreated with indicated compounds at the concentration of 10 μM for 2 h before infection of Mtb at an MOI of 20 and then at the concentration of 20 μM after washing Mtb away till harvest. Cell viability was assayed 24 hpi by measuring ATP level. (C) The combination of TNFα and type I IFN does not replace Mtb infection in inducing necroptosis. Cells are treated with indicated compounds and cytokines for 25 h and then assayed as in (A). T, TNFα, 10 ng/ml; S, Smac-mimetic birinapant, 10 μM; Z, z-VAD, 20 μM; N.I., no infection; D, DMSO; N, Nec-1, 20 μM; Q, Q-VD-OPH, 20 μM; IFN-α, 1,000 U/ml; IFN-β: 100, 500, and 1,000 U/ml. Data are means ± SD of three technical replicates and representative of two experiments. ***p* < 0.01 (two-tailed unpaired Student's *t* test).

Figure S4

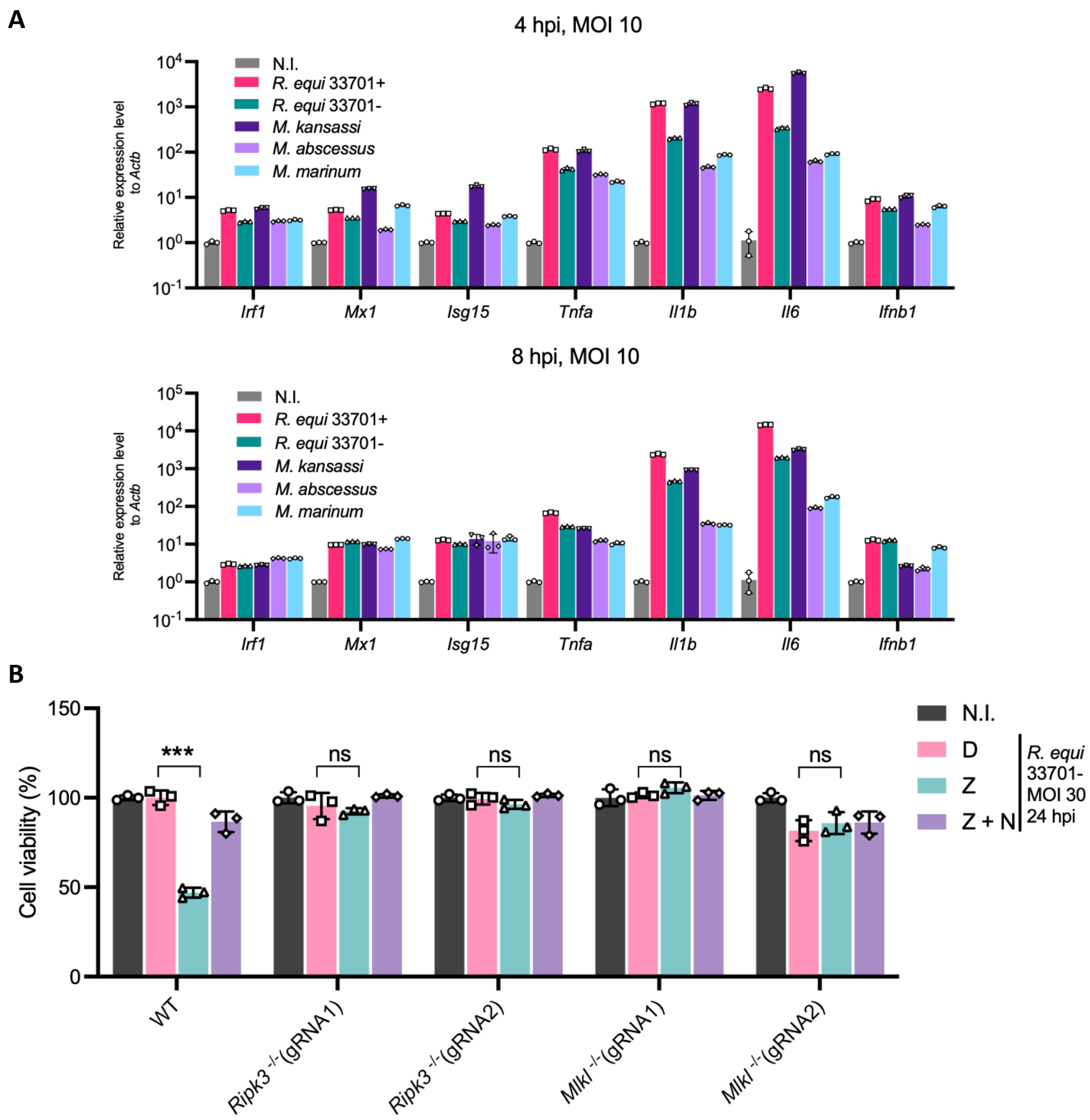

Figure S4. *R. equi* activates type I IFN signaling and induces RIPK3 and MLKL-dependent cell death in the presence of z-VAD, related to Figure 4. (A) RT-qPCR analysis on pathogen-infected BMDMs at 4 hpi (top) and 8 hpi (bottom). Total RNA was extracted and used for cDNA reverse transcription before qPCR analysis. (B) J774A.1 cells were pretreated with the indicated compounds at concentration of 10 μM for 2 h before *R. equi* infection and then at the concentration of 20 μM after washing *R. equi* away till measuring ATP level 24 hpi. Knockout cells are generated by CRISPR/Cas9–mediated targeting independently using two guide RNAs (gRNAs) for both *Ripk3* and *Mlkl*. D, DMSO; Z, z-VAD; N, Nec-1. Data are shown as means ± SD of three technical replicates and representative of two independent experiments. ns, not significant; *** $p < 0.001$ (two-tailed unpaired Student's t test).

Figure S5

A

	Exon												Intron				
Mouse	N_{126}	E	I	P	K	C	K	L	E	D	D	L_{137}	-	-	-	-	-
Casp8	AAT	GAG	ATC	CCC	AAA	TGT	AAG	CTG	GAA	GAT	GAC	TTG	GTA	AGA	CCT	AAT	CTC
Casp8-KD1	AAT	GAG	ATC	CCC	AAA	TGT	A-G	CTG	GAA	GAT	GAC	TTG	GTA	AGA	CCT	AAT	CTC
	AAT	GAG	ATC	CCC	AAA	---	AAG	CTG	GAA	GAT	GAC	TTG	GTA	AGA	CCT	AAT	CTC
Casp8-KD2	AAT	GAG	ATC	CCC	AAA	TGT	AAG	CTG	GAA	GAT	GAC	TTG	GTA	AGA	CCT	AAT	CTC
	AAT	GA-	---	---	---	---	---	---	---	---	---	---	---	---	---	-AT	CTC

B

Human	C_{203}	G	V	M	T	I	S					D	S_{211}
CASP8	TGT	GGG	GTA	ATG	ACA	ATC	TCG					GAC	TCT
CASP8$^{-/-}$(1)	TGT	GGG	GTA	ATG	ACA	ACT	TGG	AGA	GTT	CTC	CG	GAC	TCT
	TGT	GGG	GTA	ATG	ACA	ACT	TGG	AGA	GTT	CTC	CG	GAC	TCT
CASP8$^{-/-}$(2)	TGT	GGG	GTA	ATG	ACA	-CT	TCG					GAC	TCT
	TGT	GGG	GTA	ATG	ACA	-CT	TCG					GAC	TCT
CASP8$^{-/-}$(3)	TGT	GGG	GTC	CCC	TC-	---	TCG					GAC	TCT
	TGT	GGG	GTC	CCC	TC-	---	TCG					GAC	TCT

Figure S5. Genotypes of *Casp8*-KD J774A.1 and *CASP8*$^{-/-}$ U937 cells, related to Figure 5. Single clones of *Casp8*-KD or *CASP8*$^{-/-}$ cells were isolated and genotypes were verified by Sanger sequencing.

Figure S6

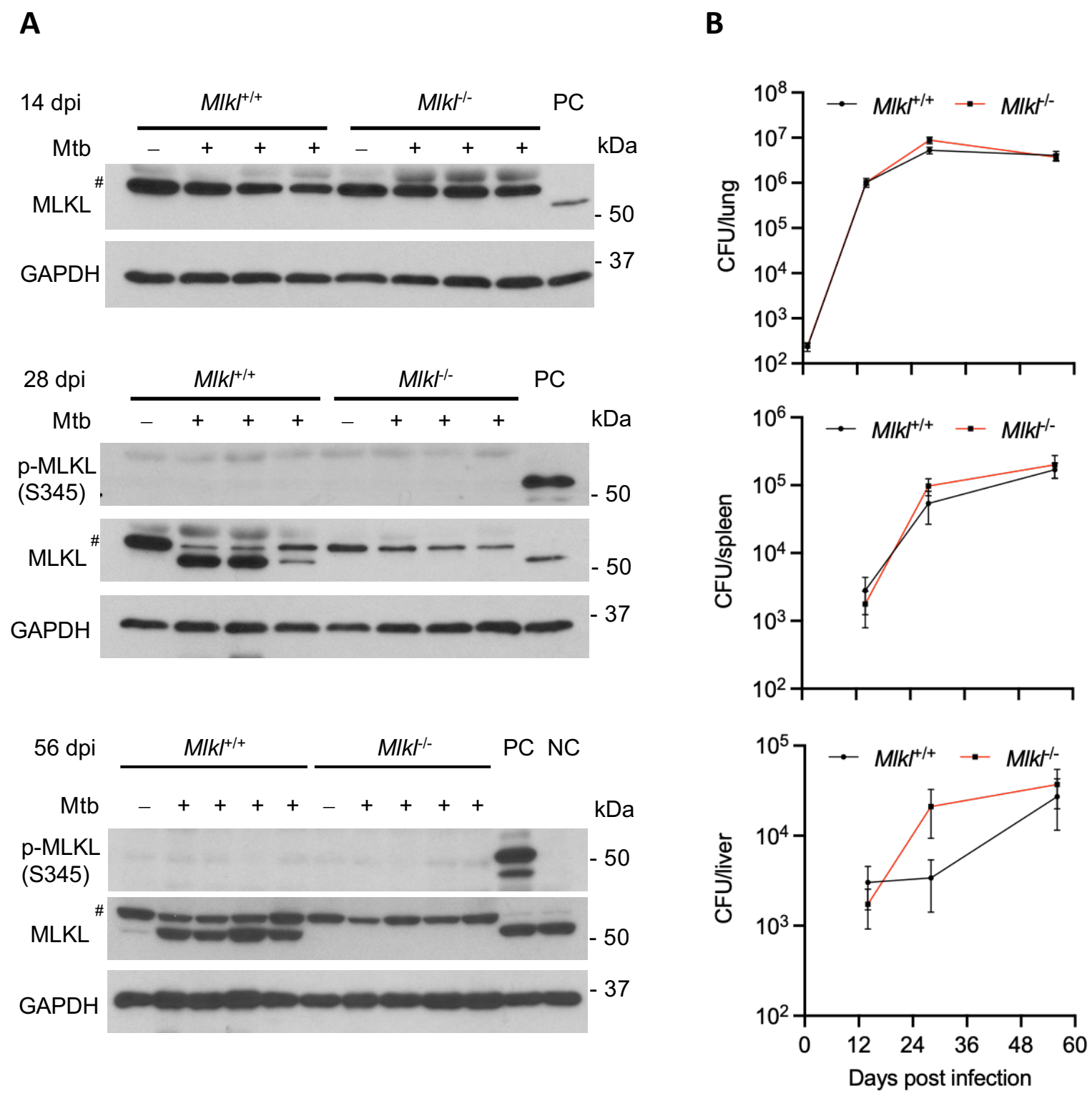

Figure S6. The absence of MLKL does not affect TB progression in Mtb-infected C57BL/6 mice, related to Figure 6. *Mlkl*$^{+/+}$ and *Mlkl*$^{-/-}$ mice were infected with 200–300 CFU of Mtb on Day 0. (A) Mtb infection on mice resulted in MLKL upregulation but not detectable MLKL phosphorylation in lung homogenates. Samples were prepared from uninfected and infected mice harvested on indicated days post infection (dpi). Each lane represents one mouse. Shown are immunoblots. PC, positive control, Mtb + z-VAD -treated BMDM lysates; NC, negative control, mock-treated BMDM lysates. #, non-specific band. (B) Mtb burdens in multiple organs of *Mlkl*$^{+/+}$ and *Mlkl*$^{-/-}$ mice. n=2 for each strain on Day 0, n=5–8 for each strain on Day 14, 28 and 56. Data are means ± SEM.

Figure S7

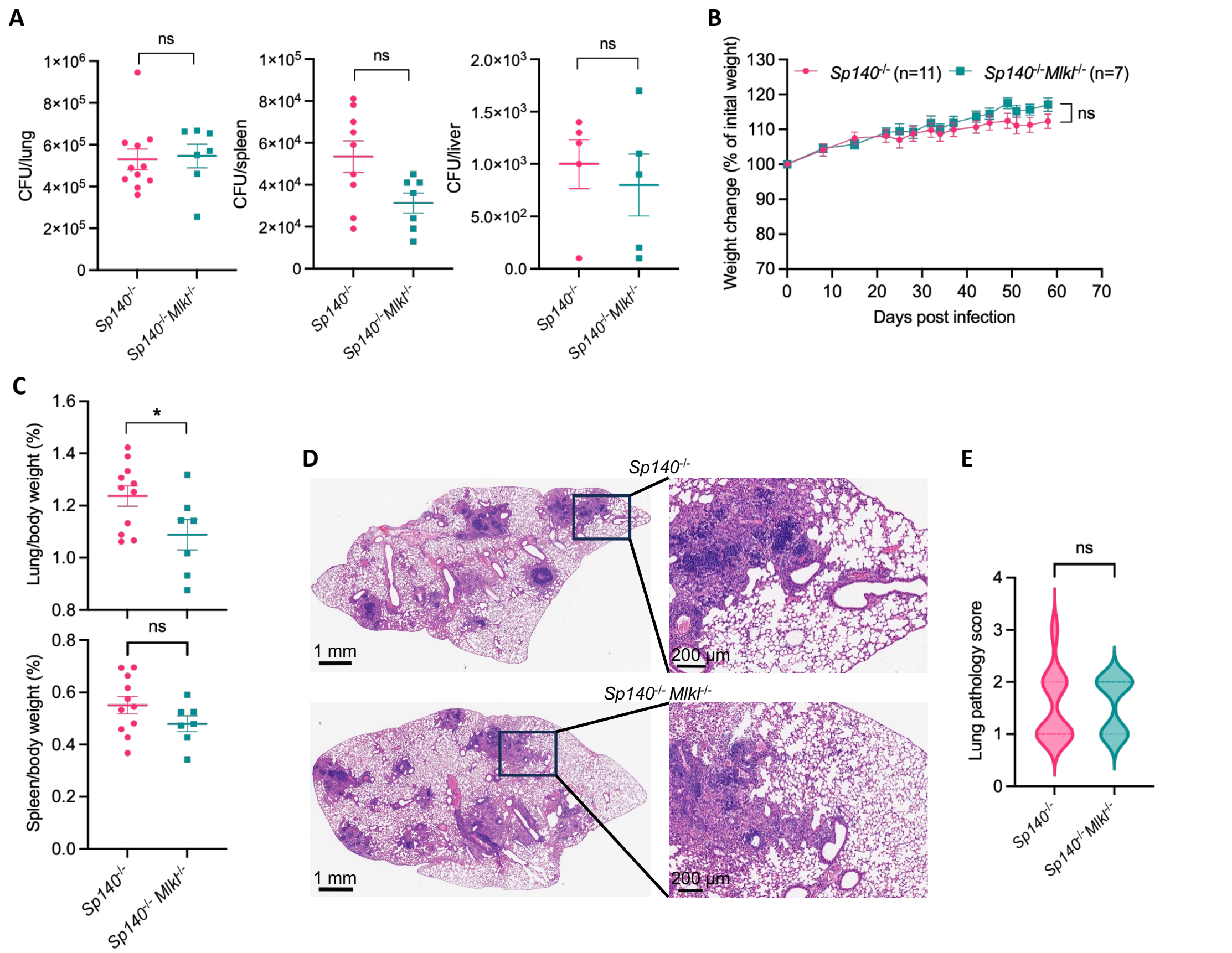

Figure S7. Medium dose of Mtb infection in *Sp140*$^{-/-}$ mice results in slow TB progression, related to Figure 6. *Sp140*$^{-/-}$ and *Sp140*$^{-/-}$ *Mlkl*$^{-/-}$ mice were infected with 400–500 CFU of Mtb on Day 0. For (A, C–E), mice were euthanized on Day 58. (A) Mtb burdens in lungs (left), spleens (middle) and livers (right) of mice. (B) Weight changes of Mtb-infected mice. (C) The ratio of lung (top) and spleen (bottom) weight to whole body weight in Mtb-infected mice. (D) Representative HE staining of lung sections of Mtb-infected mice. (E) Pathology score of lung sections of Mtb-infected mice. n=11 for *Sp140*$^{-/-}$ mice and n=7 for *Sp140*$^{-/-}$ *Mlkl*$^{-/-}$ mice. Data are means ± SEM (A-C). ns, not significant; * $p < 0.05$ (two-tailed unpaired Student's *t* test).

Figure S8

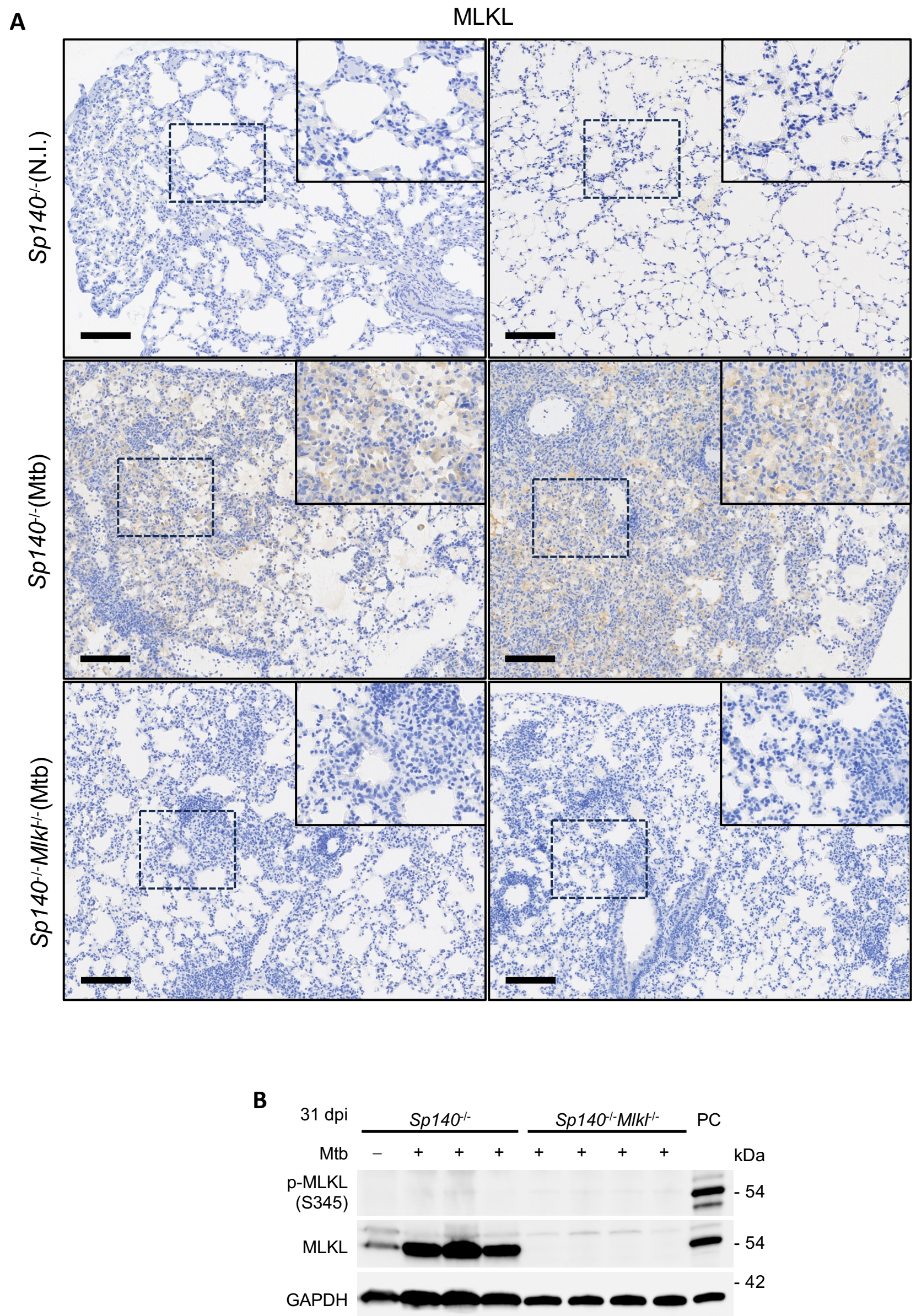

Figure S8. The induction of MLKL after Mtb infection in mouse lungs, related to Figure 6. *Sp140*$^{-/-}$ and *Sp140*$^{-/-}$ *Mlkl*$^{-/-}$ mice were infected with 4000–6000 CFU of Mtb on Day 0 and harvested on 31 dpi. (A) Immunohistochemical analysis of MLKL on mouse lungs. Images from two representative mice were shown for each group. Scale bars, 100 μm. (B) Infection on mice resulted in MLKL upregulation but not detectable MLKL phosphorylation in lung homogenates. Samples were prepared from uninfected and infected mice harvested on 31 dpi. Each lane represents one mouse. Shown are immunoblots. N.I., no infection; PC, positive control, Mtb + z-VAD -treated BMDM lysates.
